# The association between electrodermal activity (EDA), depression and suicidal behaviour: A systematic review and narrative synthesis

**DOI:** 10.1186/s12888-017-1551-4

**Published:** 2018-01-25

**Authors:** Marco Sarchiapone, Carla Gramaglia, Miriam Iosue, Vladimir Carli, Laura Mandelli, Alessandro Serretti, Debora Marangon, Patrizia Zeppegno

**Affiliations:** 1 Department of Health Sciences, University of Molise, Via Francesco De Sanctis, 1, 86100 Campobasso, Italy; 2 National Institute for Health, Migration and Poverty, Via di S. Gallicano 25/a, 00153 Rome, Italy; 3 Department of Translational Medicine, Azienda Ospedaliero Universitaria Maggiore della Carità, University of Piemonte Orientale, Via Solaroli 17, 28100 Novara, Italy; 4 Department of Health Sciences, University of Molise, Via Francesco De Sanctis, 1, 86100 Campobasso, Italy; 5 National Centre for Suicide Research and Prevention of Mental Ill-Health (NASP), Karolinska Institute, -171 77 Stockholm, SE Sweden; 6 Department of Biomedical and Neuromotor Sciences, Institute of Psychiatry, University of Bologna, Via Altura 3, 40139 Bologna, Italy; 7 Department of Biomedical and Neuromotor Sciences, Institute of Psychiatry, University of Bologna, Via Altura 3, 40139 Bologna, Italy; 8 Institute of Psychiatry, Maggiore della Carità Hospital of Novara, C.so Mazzini 18, 28100 Novara, Italy

**Keywords:** Electrodermal activity, Skin conductance, Depression, Suicidal behaviour

## Abstract

**Background:**

Electrodermal activity (EDA) and other peripheral autonomic electrical parameters have been used as indicators of emotional states, including depressive states and suicidal state. We aimed to review EDA research systematically, focusing on EDA’s usefulness as a biomarker for depression and suicidal behaviour.

**Methods:**

We searched MEDLINE, Scopus, Cochrane Library, and Web of Science databases, following PRISMA guidelines. The initial screening of articles was based on titles and abstracts; then the full text was reviewed. A preliminary synthesis of findings was developed using tables, thematic analysis and quality ratings.

**Results:**

1287 articles were screened and 77 relevant studies were identified and included in the systematic review. The studies were fairly consistent in maintaining that hypoactive electrodermal response is an established feature of patients affected by depression. There is also preliminary evidence that monitoring EDA may help to differentiate the phases of mood disorders. A few studies provided evidence that EDA can be used to differentiate acutely suicidal subjects from depressed patients who are not severely suicidal. Although EDA has been shown to be a valid, sensitive marker of suicidal ideation, suicide attempts and violent suicidal behaviour, it also seems to be influenced to some extent by antidepressant treatment.

**Conclusions:**

Most of the studies summarised in this review are quite outdated and employed a variety of designs and methods to evaluate EDA. This limits the generalisability of the results and makes it difficult to draw clear conclusions about the role of EDA in real-world settings. Electrodermal hypoactivity seems to be a reliable feature of depression and a valid marker of suicidal risk. Nevertheless, the potential utility of EDA in diagnosis, prevention, and treatment planning for depression and suicidal behaviour, should be thoroughly studied.

## Background

### Rationale

Biological abnormalities may be risk factors for depression, suicidal behaviour or completed suicide; a recent review has identified the following major categories of potential biological predictors of suicide attempt behaviour: (1) results of structural and functional brain imaging and (2) biochemical and genomic findings relating to the major neurotransmitters (serotonin, catecholamines, GABA and glutamate), the hypothalamic pituitary adrenal (HPA) axis, the inflammasome, lipids and neuroplasticity [1]. Nevertheless, there is currently a lack of biomarkers for the psychiatric field, in particular for suicidal behaviour. The prediction of suicidal risk and identification of suicidal patients will only be possible when we have an accurate picture of the interplay between biological and psychosocial factors. The use of the autonomic responses as markers of emotion, attention, decision-making, motor preparation, anticipation of reward or punishment and unconscious detection has grown considerably since the 1980s [2]. The most basic indicators of the state of the autonomic nervous system are heart rate and electrodermal activity (EDA); the former is influenced by the sympathetic and parasympathetic systems, whereas the latter is under sympathetic control only. Since a useful biomarker should be measurable in a non-invasive manner [3], the features of EDA suggest that its potential utility as a biomarker warrants careful consideration.

EDA is now the preferred term for changes in the electrical conductance of the skin, which depend on the quantity of sweat secreted by eccrine sweat glands in the hypodermis of the palmar and plantar regions [4]. Sympathetic nervous activity and variations in the sweating of the skin are regulated by environmental temperature (thermoregulatory sweating) and by central nervous activity related to affective and cognitive states (palmar, mental or emotional sweating) [5, 6]. EDA has been used as an index of emotional stimulation in several experimental studies [7].

EDA has a tonic and a phasic component. The tonic component is related to the slower components and background characteristics of the signal (skin conductance level; SCL). The phasic components are the faster-changing elements of the signal that can be associated with a stimulus (skin conductance response; SCR) or “spontaneous” or “nonspecific” (nonspecific skin conductance response; NS.SCR) [8, 9].

It has been hypothesised that the central component of EDA originates in the left hemisphere, but this lateralisation remains controversial [10]. EDA may be modulated by two different pathways: ipsilateral modulation within the limbic system, via the hypothalamus and thermoregulatory pathways and, to a lesser degree, contralateral modulation by the premotor cortex and basal ganglia [8, 11, 12].

In subjects exposed to emotional stimuli the amplitude of electrodermal response increases linearly with perceived arousal [13–16], whilst repeated presentation of identical, non-significant stimuli elicits progressively smaller reactions, a phenomenon known as habituation [8]. Individual trait differences in EDA have been observed and labelled as EDA lability. Some individuals show a high rate of nonspecific EDA and slow habituation to specific stimuli (labile individuals), whereas others show less non-specific EDA and more rapid specific EDA habituation (stable individuals) [16, 17]. EDA lability is influenced by both genetic and environmental factors [18] and is often considered dependent on trait anxiety at the individual level. It has also been proposed as an endophenotype of individual disposition towards emotional expression, self-control and inhibition of contrasting impulses [19, 20]. EDA-labiles people may be described as calm, deliberative, restrained, more good-natured, cooperative, and responsible, whereas EDA-stable people tend to be active, emotionally expressive, animated, assertive, more irritable, more antagonistic, more impulsive and more irresponsible [21, 22]. Both depression and suicide have been extensively investigated from a physiological point of view, and the extant research has consistently demonstrated that depressed and suicidal patients show electrodermal hypoactivity and can therefore be described as EDA-stable [23–26].

To the best of our knowledge the first review of EDA in depression was published by Straub et al. in the early 1990s [25]. It acknowledged that electrodermal hypoactivity had been repeatedly associated with affective disorders, but raised doubts about whether EDA could be considered a marker of depression, pointing to conflicting results and differences in laboratory conditions and suggesting that the impact of person-situation-environment dynamics on EDA had been underestimated. A more recent review of galvanic skin response (GSR) confirmed that patients with mood disorders show low or flat EDA profiles, but the authors pointed out several limitations in the evidence, including that many of the EDA studies are quite old and were conducted using outdated methods and technology [26]. The Vahey & Becerra review [26] anyway focused specifically on mood disorders and yielded a smaller number of eligible studies (41 vs. 77).

A summary of the current knowledge about the relationships between EDA and depression and between EDA and suicidal behaviour is timely, given the lack of recent comprehensive reviews [24] and the recent publication of the study protocol for a multi-centre, naturalistic, clinical study of electrodermal orienting reactivity in a large sample of adult depressed patients [27].

### Objectives

Our objective was to carry out a systematic review of studies investigating:

1. The association between EDA and depression (research comparing depressed patients and controls, depression subgroups, depressed patients and other psychiatric patients, elicitation methods and EDA, EDA and hormones, EDA and antidepressants and other studies of EDA characteristics).
2. The association between EDA and suicidal behaviour (including research on suicidal risk, suicidal ideation only, completed/attempted suicide, violent/non-violent suicidal behaviour, impulsive/non-impulsive suicidal behaviour and suicide prevention) in depressed patients.

## Methods

### Selection of studies

The inclusion criteria were the following:

- EDA was measured.
- Design: randomised controlled trial (RCTs), quasi-experimental (e.g. non-randomised controlled studies and before-and-after studies), observational or meta-analytical.
- Participants of any age or gender in a community or clinical setting.
- The following types of study were eligible for inclusion:
  - studies involving participants with a primary diagnosis of mood disorder (unipolar depression, bipolar disorder, dysthymia, cyclothymia) who were in a depressive phase;
  - studies investigating the effects of an antidepressant compound on EDA;
  - studies involving participants with suicidal ideation or suicidal behaviour (suicide attempts, completed suicide).

Exclusion criteria were:

- Primary focus on psychiatric disorders other than mood disorders.
- Investigation of the effects of EDA on medication other than antidepressants.
- Case report, letter to the editor, conference paper, dissertation, personal opinion or commentary.
- Papers not written in English.

### Data sources and search strategy

We carried out an electronic literature search to identify relevant studies. PubMed, Scopus, Cochrane Library and Web of Science were searched from the inception of the databases up to 12^th^ April 2017. The search strings used in each search engine are reported in the Appendix 1. Articles were selected in accordance with the Preferred Reporting Items for Systematic Reviews and Meta-Analyses (PRISMA) diagram [28, 29]. The selection was done by three reviewers (M.S., M.I., V.C.) who independently selected titles, abstracts and full-text publications according to the inclusion and exclusion criteria described above. In the first stage of the selection process titles were screened to exclude those that were clearly not relevant to the review and then each reviewer read the abstract and full text of the remain articles and selected the relevant ones. Disagreements between reviewers were resolved through group discussion.

The following information was extracted from all publications: country, design, characteristics of study participants (number of subjects, diagnosis, mean age, % women), EDA variables and summary of main study findings. Regrettably, availability of information on several issues - sample and diagnosis; study design and protocol; type of EDA assessment; type of data presented in the results etc. - was very uneven and this meant that neither meta-analysis nor quantitative synthesis was possible, nor was it possible to adhere completely to the “Guidance on the conduct of narrative synthesis in systematic reviews”; nonetheless the guidance on data analysis and presentation was followed as closely as possible [30]. Study quality was appraised, where applicable, using the Newcastle-Ottawa Scale (NOS) [31].

## Results

**Fig. 1:**
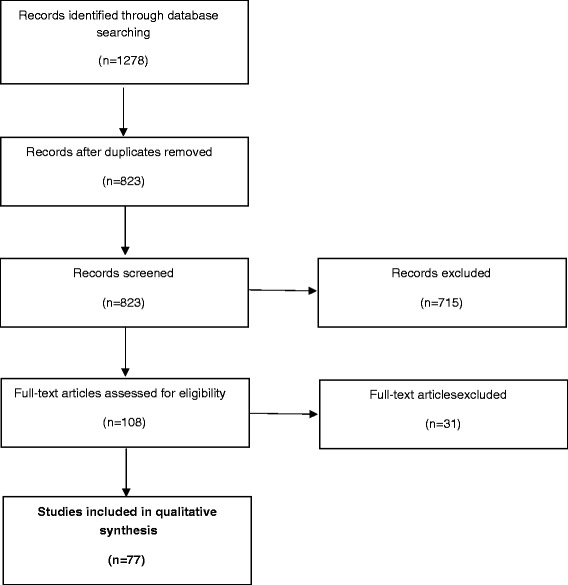
PRISMA Flowchart

The electronic searches identified 1278 studies; once duplicates removed 823 records remained of which 606 were excluded because the title clearly had no relevance to the review; 109 records were excluded based on the Abstract; a further 68 studies were excluded because they were not written in English and 41 studies because the full text was not available. We assessed the full text of 108 articles and excluded 31 studies at this stage. The main reasons for exclusion were: study population did not match inclusion criteria (no primary clinical diagnosis of depression), focus on psychophysiological variables other than EDA (e.g. heart rate), basic, non-clinical research on EDA and wrong type of publication (conference session; letter to the editor; case report). Seventy-seven studies met all the inclusion criteria and were included in this review. The selection process is represented in Fig. 1.

Summaries of all the studies are presented in Tables 1 and 2 (EDA and depression) and 2 (EDA and suicidal behaviour).

**Table 1:** Summary of selected studies – Electrodermal activity (EDA) and suicidal behavior

AUTHORS (COUNTRY AND YEAR)	SUBJECTS (DIAGNOSIS; AGE; MALE/FEMALE) AND CONTROLS (AGE; MALE/FEMALE)	MEASURES	MAIN STUDY FINDINGS	STUDY DESIGN (QUALITY^1^)
Crowell et al. (USA, 2005)	− 23 parasuicidal adolescent girls, age 14–18, − 23 age-matched controls.	EDA recordings during the last minute of a 10-min resting baseline and for 10 min of recovery following the end of a sad movie.	No significant differences on measures of EDR between parasuicidal girls and healthy controls.	Case-control(3)
Edman et al. (1986)	35 drug-free, suicidal inpatients (mean age 41 years; 63% females): − 24 inpatients after a suicide attempt; − 11 patients with suicidal ideation.	SCL and habituation of the SCR.	No differences in the SCL. Tendency to lower figures in NS.SCR in patients who used violent suicide methods. All violent attempters (at baseline and follow-up) were fast habituators. Lowest frequency of fast habituators in patients with suicidal ideation.	Cross-sectional
Jandl et al. (Germany, 2009)	MDD inpatients divided in 3 groups: − 16 with history of ‘hard attempted suicide’ (e.g., hanging, stabbing) (47.3±11.9 years; 56% females); − 16 with a history of ‘soft attempted suicide’ (e.g. poisoning) (49.8±8 years; 69% females); − 18 no attempted suicide (47.9±11.6 years; 67% females).	NS.SCR frequency, SCR amplitude and EDA Habituation Rate measured during an auditory habituation paradigm.	Significantly lower EDA habituation rate in both suicide attempters groups. No difference among the 3 groups in SCR amplitude or NS.SCR frequency. Women showed faster habituation.	Cross sectional
Keller et al. (Germany, 1991)	− 23 patients with suicide attempt divided into violent and non-violent method (violent, N=14, 41.4±9.5 years, 64% females; non-violent, N= 9, 37.2±9.7 years, 66.6% females). − Age and sex matched groups (non-suicidal depressed patients/patients with suicidal thoughts.	Number of stimuli until habituation, nonspecific SC reactions, height of first amplitude and SCL measured during an habituation experiment.	All patients who had used a violent method and patients who committed suicide in the year following the experiment were fast habituators. No difference in EDA between non-suicidal patients, those with suicidal thoughts or suicide attempts.	Case-control (4)
Sarchiapone et al. (Europe, 2017)	-1573 patients with a primary clinical diagnosis of depression, either currently depressed or in remission.	− ElectroDermal Orienting Reactivity (EDOR Test)	It is hypothesized that the EDOR Test will identify electrodermal hyporeactive depressed patients with a high suicidal proneness. Patients who reveal strong death intent are expected to be hyporeactive at the EDOR test, in most cases; non-hyporeactive patients are expected to have suicide attempts with strong death intent in a minority of cases.	Multicentric/ Study protocol
Spiegel (US, 1969)	Inpatients including: - 16 controls with no history of suicide threats or attempts and denying current suicidal thoughts; - 15 inpatients with suicide threats or preoccupations but no history of attempts; - 13 attempters with a history of one or more suicide attempts.	GSR First two sessions: word association. 4 days later, a series of words at irregular intervals, recording skin resistance changes.	Relatively low reactivity of threateners might be a function of depression. Threateners were the least reactive in terms of GSR to the word “suicide” Significant positive association between magnitude of GSR and intensity of affective meaning assigned to the concept “suicide” in all patients.	Crossover
Thorell (Sweden, 1987) Thorell et al. (1987a) Thorell et al. (1987b)	− 59 in- and out-patients with major depressive episode and dysthymic disorders (42±13.2 years; 54% females); − 59 mentally and somatically healthy subjects (42.4±13.3 years; 54% females)	SCL at the onset of the first stimulus, mean SC fluctuation rate (SCFr) per minute, SCR magnitude (SCRm) to the first stimulus, SCR rate (SCRr), and index of SC nonresponding (SCRi).	Significantly lower central SCL, SCRm, SCRr, and SCRi, but not SCFr and habituation values in patients than in controls. Considering medications, no statistically significant difference in any EDA variable. No significant difference in any EDA variable among dysthymic disorder vs major depressive episode and melancholic vs non-melancholic major depressive episode. Patients with endogenous depression had significantly lower SCL, SCRr, and SCRi. Patients with a high level of inhibition showed significantly lower SCL and SCRi. Suicidal behavior related to low electrodermal responsivity (EDR) and low stimulus-unrelated phasic activity, but unrelated to tonic EDA. Extreme hyporesponsivity in the suicide attempters was observed irrespective of whether the attempt had been made previously or during the current depression.	Case-control (5)
Thorell et al. (2009)	− 279 depressed patients − 59 healthy subjects	Habituation of the electrodermal response	Sensitivity 96.6% and specificity 92.9% of electrodermal hyporeactivity for suicide and 83.3% and 92.7%, respectively, for suicide and⁄or violent attempts	Meta-analysis
Thorell et al. (Germany, 2013)^2^	783 depressive patients (126 bipolar patients; 539 unipolar patients; 118 with other diagnoses) (42.9±11.5; 58% females): − 32 violent suicide; − 4 nonviolent suicide; − 84 violent suicide attempt; − 103 nonviolent suicide attempt; − 560 other or no suicidal behavior.	Habituation of electrodermal responses measured during an auditory habituation paradigm.	Prevalence of electrodermal hyporeactivity was high and highest (80%) among bipolar patients and was independent of severity of depression, trait anxiety, gender and age. Sensitivity and specificity for suicide, and for suicide and violent suicide attempt were 83%, 98% and 74%, 88%, respectively. Hyporeactivity was stable, while reactivity changed into hyporeactivity in a later depressive episode.	Cohort (7)
Wolfersdorf et al. (Germany, 1996)	− 11 patients with personality disorders who attempted suicide (23±4 years; 100% females); − Depressed non-suicidal patients (100% females); − Depressed suicide attempters (100% females); − Schizophrenic non-suicidal patients (100% females); − Schizophrenic suicide attempters (100% females).	SCL, number of NS.SCR, habituation of the SCR	Significant differences in EDA values between women with personality disorders who attempted suicide and non-suicidal women suffering from depression. No difference between personality disorder and depressive patients who attempted suicide . No significant differences in EDA values in a habituation study between personality disorder patients and schizophrenic women with or without histories of suicide attempts.	Cross-sectional
Wolfersdorf et al. (Germany, 1999)	− 30 depressed violent suicide completers (mean age 43.8 years; 57% females); − Age and sex matched non-suicidal depressed patients; − Age and sex matched depressed patients with suicidal ideations; − Age and sex matched depressed patients with suicide attempts.	Number of stimuli up to habituation (habituation score, HS), SCL and amplitude of SCR after the first stimulus measured during a habituation experiment using auditory stimuli.	Depressed patients who died by suicide showed significantly lower HS, first amplitude of the SCR and SCL than non-suicidal depressed patients. Suicides showed a significant lower HS than non-suicidal patients or depressive patients with suicidal ideation. No significant difference between suicide and suicide attempters groups.	Case-control (5)

Legend:

^**1**^The “Newcastle-Ottawa Scale (NOS) for assessing the quality of case-control and cohort studies” was used to evaluate the case-control (scores 0-10) and cohort studies (scores 0-12)

^2^The sample comprises patients treated on the Depression Ward of the Centre for Psychiatry, Weissenau in southern Germany between 1985 and 2002. The studies of Keller et al. (1991), Wolfersdorf et al. (1996) and Wolfersdorf et al. (1999) were conducted in the same centre

EDA = Electrodermal Activity; EDOR = ElectroDermal Orienting Response; HS = Habituation Score; MDD = Major Depressive Disorder; NS.SCR = Nonspecific Skin Conductance Response; SCL = Skin Conductance Level; SCF = Skin Conductance Fluctuation; SCR = Skin Conductance Response

**Table 2:** Summary of selected studies – Association between EDA and depression

AUTHORS (COUNTRY AND YEAR)	SUBJECTS (DIAGNOSIS; AGE; MALE/FEMALE) AND CONTROLS (MEAN AGE; MALE/FEMALE)	MEASURES	MAIN STUDY FINDINGS	STUDY DESIGN (QUALITY^1^)
Barg et al. (Germany, 1996)	− 16 depressed patients (36.1±1.9 years; 50% females), no medication; − 16 depressed patients (45.8±10.2 years; 50% females) treated with paroxetine; − 16 depressed patients (45.4±9.9 years; 50% females) treated with imipramine.	SCL, non-specific SCR, SCR	Patients treated with imipramine showed a higher heart rate and lower EDA than the other two groups.	Case-Control (5)
Bernstein et al. (US, 1988)	− 50 schizophrenic patients (34.8±9.5; 30% females); − 50 depressive patients (32.5±9.7 years; 66% females); − 50 controls (27.3±7.6 years; 58% females).	SCR recorded during auditory stimuli presentation. A subsample of each group was told they needed do nothing during tone presentations (habituation series); other subsamples had to press a pedal for each designated target signal, ignoring all nontarget tones.	No differences between hands in SCR for any diagnostic group Compared to normal controls, schizophrenic patients showed higher nonresponding in the habituation series, but no difference when target tones were presented. Depressives were equally nonresponsive during the habituation series, but they did not show a decrease in SCR nonresponding to the target tone.	Case-control (4)
Bernstein et al. (US, 1995)	− 69 schizophrenic patients (34.9±8.5 years; 42% females); − 45 mood disorder patients (36.6±10.1 years; 62% females); − 67 normal controls (31.2±10.1 years; 49% females).	SCR recorded during exposure to tones.	Schizophrenic and depressed patients were significantly more often classified as non-responders. The proportion of non-responders did not vary with neuroleptic or antidepressant medication.	Case-control (4)
Bob et al. (2011)	− 44 unipolar depressed outpatients, (37.06±8.72); − 35 healthy controls (35.12±8.10; 57% females).	EDA measured bilaterally.	Absence of differences between EDA in relapse and in remissions as well as between pharmacotherapy response and EDA.	Case-control (4)
Bonnet et al. (France, 2004)	− 9 non-depressed Chronic Low Back Pain (CLBP) patients, (43 years; 55% females); − 9 depressed CLBP patients, (46.1 years; 55% females); − 9 depressed controls free of pain (DCs), (44.1 years; 55% females); − 9 healthy controls free of pain and depression, (45.3 years; 55% females).	EDA recorded during a 3-min rest period and during a subsequent stimulation period. Stimuli were 3 identical pure tones.	Non-depressed patients presented an increased EDA, especially a higher frequency of non-specific fluctuations, than the 3 other groups. SCL was lower in the 2 groups of depressed participants than in the healthy control group.	Case-control (4)
Brankovic (Serbia, 2008)	− 57 patients with major depressive disorder and currently in depressive episode (43.1±10.9 years; 56% females); − 52 healthy controls (39.8±8.1 years; 60% females).	5 parameters of the SCR recorded when reading emotional eliciting stories.	Initial SCR was larger in controls than depressed patients. Positive feedback loop was greater in depressed patients than controls. Negative feedback loop was stronger in depressed patients than controls. Length of time needed for the magnitude of SCRs to decrease to half of the original response magnitude was longer in depressed patients than controls.	Case-control (3)
Breyer-Pfaff et al. (1982)	37 patients with primary depressive disorders (22 endogenous and 15 non endogenous) (81% females) treated with amitriptyline.	SCLs, SF, habituation and amplitude of the SCR recorded during the exposure to tones and during active and passive situations. Measures repeated on the day before treatment began and again after 14 and 28 days of treatment.	Compared to baseline levels, EDA state at day 14 can be considered inhibited and labile (larger phasic and tonic reaction amplitudes). After 4 weeks of treatment, lability had returned to baseline, but inhibition was unchanged. No significant correlation between EDA measures and plasma drug levels.	Case-series
Byrne (Australia, 1975)	− 18 acute depressive inpatients (20-60 years; 50% females): − 10 “neurotic depressive”; − 8 “psychotic depressive”; − 11 normal controls (24-45 years; 45% females).	SCR amplitude, NS.SCR, habituation rate.	Higher mean SCR, higher frequency of NS.SCRs, and slower skin response habituation rata in the neurotic depressive sample. Higher mean SCR amplitude in the control sample. No significant difference between skin response habituation rates of either the control and neurotic depressive, or control and psychotic depressive samples.	Case-control (1)
Carney et al. (US, 1981)	− 15 inpatients with primary affective disorder (32±6.9 years; 100% females) − 15 age-and race-matched controls (31.5±7.5; 100% females)	SCL	Significantly lower SCL in depressed subjects. No significant correlation between SCL and measures of anxiety or depression for patients with primary affective disorder. Significant correlations between a measure of depression and SCL in normal controls.	Case-control (5)
Dawson et al. (US, 1977) Dawson et al. (1985)	− 20 hospitalized depressed patients (63.8±7.8 years; 80% females); − 20 non-depressed controls (62.2±7.6 years; 80% females).	SCL and SCR recorded at rest and during a variety of tasks before and after a series of electroconvulsive shock treatments (ECTs)	Depressed patients, compared to non-depressed controls during the pre-ECT test, exhibited lower SCLs, smaller phasic SCRs with longer latencies. Little EDA changes following ECT. Overall efficiency of SCL was of 70% and of SCR was 80%.	Case-control (4)
Donat & McCollough (US, 1983)	− 10 chronically depressed subjects (18-25 years; 100% females); − 10 controls (18-25 years; 100% females).	SCL	SCL correctly identified 9 of the 10 depressed group subjects and 7 of the 10 control group members under resting conditions. No difference between depressed subjects and controls under conditions of laboratory stress and of imaginal-role stress. SCL reliably identified group membership, with lower levels in the depressed group.	Case-control (4)
Falkenberg et al. (Germany, 2012)	− 16 patients with MDD (37±15 years; 50% females); − 16 healthy controls (35±14 years; 50% females).	SCR recorded during standardized mood induction using happy and neutral pictures and funny and neutral cartoons.	MDD patients had higher SCRs in the cartoon condition than controls. Women revealed lower overall SCRs compared to men.	Case-control (5)
Fraguas et al. (US, 2007)	8 unmedicated patients diagnosed with MDD (35±12 years; 62,5% females) treated with fluoxetine 20 mg per day for 8 weeks.	SCL and SCR measured at basal condition and during four induced emotional states: happy, angry, sad and neutral.	Significant positive correlations between the percentage reduction in depression scores and increases in SCR only during the neutral emotion condition.	Case-series
Giedke et al. (Germany, 1980)	− 18 patients with primary depression (15 unipolar, 3 bipolar) (46.2±13.1 years; 83% females); − 27 age-matched healthy controls (45.1±15.4 years; 70.4% females).	GSR/SRR (Skin Resistance Responses). Response condition: participants were asked to react to the second of two identical tone stimuli.	In the experimental response condition controls increased their number and amplitude of SRRs to tone-stimuli more than patients. No significant correlation between GSR and both self-rated and physician-rated psychopathology.	Case-control (5)
Giedke & Heimann (1987)	− 59 drug free patients with primary MDD (47±11 years; 69% females) assigned to a double-blind treatment with amitriptyline or oxaprotiline; − 30 healthy controls matched for age and sex.	SR and habituation of SR orienting response.	Patients with primary MDD exhibited significantly fewer spontaneous fluctuations of SR and a faster habituation rate of SR orienting response. SR level did not differ between groups. Most patient/control differences remained unaffected irrespective of the drug applied.	Case-control + RCT (5)
Greco et al. (Italy, 2014)	− 10 patients affected by bipolar disorder I or II; − 10 healthy subjects (20-32 years; 50% females).	Tonic and phasic features of EDA measured during an emotional elicitation protocol.	Phasic features well discriminated among depression, mixed state, and euthymia. Mixed-state shows a strong tonic hypoactivity compared to euthymic state. Healthy subjects showed no statistical difference on each of the EDA feature patterns.	Cross-sectional
Hattangadi et al. (Canada, 1968)	- 263 psychiatric inpatients, including 51 depressed patients (27 neurotic and 24 psychotic depression); - 58 healthy controls.	GSR	Patients responded less than controls to the indifferent stimuli. No significant difference in response frequency between patient categories. No significant difference in latency, either between normal subjects and patients, or between any two diagnostic categories. All psychiatric patients in all categories gave lower amplitude responses than the normal group. No significant difference between medicated and non-medicated patients in any diagnostic group as far as GSR frequency and latency. Patients receiving antidepressant medication had higher amplitudes than those receiving anti-psychotic medications.	Case-control (4)
Have et al. (Iceland, 1991)	− 21 patients with Alzheimer dementia (80.6 ± 9.8 years); − patients with depression (75.9 ± 7 years); − 19 healthy volunteers (79 ± 11.4 years).	SCL	No difference in SCL among patients' groups. Very low SCL in the overall sample, likely due to a decline in the number of active sweat glands in old people and diminished sweat production.	Case-control (3)
Heimann (Germany, 1978)	− 95 depressive and depressive-anxious patients; − 18 of these 95 treated with amytriptylin for 4 weeks; − 27 of these 95 retested at a 1-year follow-up.	SRR, SRL (Skin Resistance Level). Orienting reaction and habituation tested after two minutes of rest, with a series of tones and of flashes. Assessment of changes in the dimension of activation after 14 and 28 days of treatment with amytriptylin, and 1-year follow up after the first examination during a depressive state.	Patients in the activated and inhibited cluster showed the smallest changes at follow-up, whereas all four patients of the labile-activated cluster change to another group. Preponderance of cases with lower activation after treatment with amitriptylin.	Cross-sectional
Iacono et al. (1983) Iacono & Tuason (1983)	− 26 unipolar depressed patients (37.8±12.7 years; 23% females); − 24 with bipolar affective disorder (36±12 years; 33% females); − 46 healthy control patients (35±11.9 years; 17.4% females).	SCL.	The EDA of affective disorder patients was uniformly depressed across all tasks and conditions. No consistent bilateral asymmetries in EDA were observed.	Case-control (5)
Iacono et al. (1984)^2^	− 22 unipolar outpatients in remission (41±12.7 years; 77% females); − 22 bipolar outpatients in remission (38.5±14.4 years; 68% females); − 26 controls (41.3±12.2 years; 88% females).	SCLs and SCRs recorded during blowing up a balloon until it burst and during the exposure to tones, and eight familiar sounds.	Compared to the control subjects, the affective disorder patients (especially the unipolar patients) responded significantly less to the balloon task, the highest tones, and the familiar sounds, had lower tonic levels and a larger proportion of them failed to respond to the stimuli. Several measures of EDA displayed moderately high one-year retest stability.	Case-control (3)
Ikeda et al. (Japan, 1982)	20 healthy volunteers (23.1 years; 0% females): − Amitriptyline 30 mg/day, 3 times, for 3 days; − Nomifensine 100 mg/day, 2 times, for 3 days; − Placebo 3 times, for 3 days.	SCR recorded before the experiment and 8 hours after the final dose.	In the amitriptyline group, the NS.SCR decreased. In the nomifensine group, NS.SCR tended to increase.	
Kamenskaya & Mikhailova (Russia, 1985)	− 50 manic-depressive patients in the depressive phase prior to treatment (20-45 years), divided into 3 groups according to the nature of the principal affect (anguish, anxiety, or apathy); − 25 healthy individuals (20-45 years).	SCR in the background and during the presentation of indifferent stimuli (opening the eyes, light flashes) and in a stress situation.	Depressed patients had an increased latency and lower amplitude of the SCR than healthy subjects. The absence of a SCR was observed in 20% of patients, more often with a dominant anxiety affect.	Case-control (2)
Lader & Wing (UK, 1969)	− 35 inpatients with a primary diagnosis of moderate or severe depression: − 17 Predominantly agitated (45.8 years; 59% females); − 13 Predominantly retarded (42.3 years; 69% females); − 5 Uncomplicated depressives (30.2 years); − 35 age and sex matched controls.	SCLs, SCRs, NS.SCRs and habituation rate recorded during exposure to tones.	Agitated patients and retarded patients had, respectively, significantly higher and lower mean SCL than controls. Agitated depressives had the highest conductance levels at the end of the recording session than at the start. Healthy subjects were the most reactive ones, and habituated significantly more rapidly than did the agitated depressives. Retarded patients gave a significant lower number of SCRs than agitated patients. The agitated patients had significantly more and the retarded patients had significantly less fluctuations than the normal subjects.	Case-control (4)
Lapierre & Butter (Canada, 1978)	− 20 agitated depressed patients (mean age 38 years and 2 months; 75% females); − 20 retarded depressed patients (mean age 37 years and 8 months; 70% females).	Spontaneous GSR was monitored for 5 minutes. The patient was then given a series of 6 randomized visual and/or auditory stimuli, and the latency period for a EDA response and the magnitude of the response was assessed. Then: Maprotiline and imipramine administration, double-blind. Measurement were repeated after 3 hours, 4, 7, 14 and 28 days.	Basal skin resistance was significantly lower for the agitated depressive. The GSR response was consistently greater in the agitated depressives than in the retarded. Gradual but nonsignificant increase in skin resistance and reduction in the magnitude of the GSR response as treatment progressed in both drug groups. Still the agitated depressives maintained more reactivity on this parameter. No major difference in GSR was found between imipramine and maprotiline.	Double blind controlled study
Lapierre & Butter (Canada, 1980)	− 20 agitated depressed patients (mean age 38 years; 75% females); − 20 retarded depressed patients (mean age 38 years; 70% females); − 20 volunteer controls (mean age 24 years; 75% females).	SCL and SCR recorded prior, during and after visual and auditory non-signal sensory stimulations and a reaction time stimulation.	SCLs significantly higher for both depressed groups. Depressed patients had longer latency time of onset of an EDA response than in volunteers.	Case-control (0)
Lemaire et al. (France, 2015)	− 45 bipolar outpatients: − 15 remitted (41.67±10.19 years; 47% females); − 15 moderately depressed (43.4±11.65 years; 80% females); − 15 moderately manic (41.53±8.13 years; 73% females); − 101 healthy controls (43.86±11.96 years; 49% females).	Maximum SCR amplitude recorded following the presentation of affective and neutral pictures during passive viewing and during experiential suppression.	Negative and positive pictures elicited SCRs of similar maximum amplitudes, greater than those elicited by neutral pictures. The maximum SCR amplitude was reduced during experiential suppression in all groups but this effect was more pronounced in depressed patients.	Case-control (4)
Levinson (US, 1991)	− 36 schizophrenic patients (31.4±6.9 years; 36% females); − 17 patients schizoaffective disorder (30.3±9 years; 59% females); − 24 inpatients with MDD (psychotic or nonpsychotic), or the mainly affective subtype schizoaffective disorder (36.9±12.4 years; 54% females); − 25 control subjects (30.6±9 years; 44% females).	SCL, SCR, NS.SCR, SC non-response, habituation score, reaction time recorded during four paradigms: − a series of tones in a no-task habituation paradigm; − a similar series of higher tones; − a series of tones with a button-press (reaction time) task; − a loud white noise stimulus (without task).	Schizoaffective subjects were more likely to be non-responders, and had lower (faster) mean habituation scores than other groups. Schizophrenic, depressed and controls subjects had similar mean habituation scores and proportions of non-responders. Depressed subjects showed some evidence of autonomic hyperarousal (higher tonic SC level, trend toward more NS.SCRs).	Case-control (4)
Lewinsohn et. al (USA,1973)	Two studies on the same sample composed by − -12 depressed patients; − -12 psychiatric control patients; − -12 healthy control subjects.	Autonomic response (skin resistance) to aversive stimulation and adaptation over repeated presentations of the same aversive situation.	In both studies, the depressed group was found to be more responsive to the aversive stimulus and the overall SCL was highest for the depressed subjects.	Case-control (5)
Lindsey et al. (US, 2011)	- 24 currently depressed Seasonal Affective Disorder (SAD) participants (41.58±11.72 years; 92% females); - 24 demographically-matched controls with no depression (41.83±12.75; 92% females).	SCL, surface facial electromyography and self-reported emotional responses to light- and season-relevant stimuli Task: 30 digital photographs of 5 scenes presented under 6 conditions (light intensity – 2 conditions; seasonal cues – 3 conditions).	SAD participants displayed more frequent SCR, greater SCR magnitude, and more self-reported depressed mood in response to overcast stimuli and lower SCR magnitude, and less self-reported depressed mood in response to sunny stimuli.	Case-control (5)
Mardaga & Hansenne (Belgium, 2009)	− 20 subjects with MDD (22-59 years; 55% females); − 20 healthy controls (24-59 years; 55% females).	SCR recorded following the presentation of neutral, pleasant, and unpleasant pictures.	Pleasant pictures elicited more and larger responses than unpleasant ones in control but not in depressed subjects. Depressed subjects showed generally faster half-recovery times.	Case-control (6)
Mestanikova et al. (Slovak Republic, 2015)	− 25 depressed adolescents (14.6± 0.4 years; 52% females); − 25 age/gender matched healthy controls.	EDA measured in sitting and supine position, at rest.	EDA diminished in the supine resting position in MDD patients compared to healthy controls.	Case-control (4)
Miquel et al. (Spain, 1999)	− 27 depressed outpatients (4 bipolar disorder in the depressive cycle; 17 MDD; 5 depressive neurosis; 1 subject non-specified depressive disorder) (34.63±10.05 years; 19; 70% females); − 27 normal subjects (33.44±10.27 years; 63% females).	SCL and SCR recorded during a series of tones.	Depressive patients displayed lower basal SCLs and lower conductance amplitudes to the first stimulus and to stimulus change. A trend towards habituation was detected in both groups, but neither difference between them, nor differences in habituation speed were found between depressive and healthy subjects.	Case-control (2)
Mirkin & Coppen (1980	− 13 depressive inpatients (58.6±1.9 years; 72% females). − 15 normal controls (54.2±1.7 years; 60% females).	SCL and SCR recorded during exposure to tones.	Higher proportion of electrodermal non-responders in patients than controls. Endogenous depressive patients had significantly lower SCLs than either the non-endogenous patients or controls.	Case-control (1)
Myslobodsky & Horesh (Israel, 1971)	− 10 endogenous depressive patients (51±6.79 years; 70% females); − 9 reactive depression patients (43±6.29 years; 89% females); − 14 normal subjects (48±17.59 years; 57% females).	EDA recorded at rest and during three experimental conditions: a visual-imagery task, a verbal task, a neutral tone habituation sequence.	In endogenous depression EDA was higher on the left hand compared with the right under all the conditions studied. In reactive depression EDA was higher on the left side during the verbal task and tone habituation sequence and on the right side in the visual task.	Case-control (3)
Nissen et al. (2010)	− 23 patients with MDD; 35 healthy control matched for sex, age, and IQ.	SCR recorded during the presentation of Conditioned (CS+) and non-Conditioned (CS-) Stimuli.	MDD patients responded stronger to the CS+ than to the CS-.	Case-control (4)
Noble & Lader (UK, 1971)	− 34 inpatients with a primary diagnosis of depression (12 males, 44.3±13.6 years; 22 females, 35.7±11.4 years); − Subgroups of 10 agitated and 10 retarded depression patients.	SCLs, SCRs, NS.SCRs recorded at rest and during mental arithmetic, before and after ECT.	The stress of mental arithmetic was associated with increase in skin conductance. The difference between the basal and stress recordings (reactivity) was highly significant. Reactivity was reduced after ECT. Low skin conductance correlated significantly with severity of depression. A reduction in NS.SCR correlated with high scores for depressed mood and retardation. Before ECT the agitated patients had significantly more fluctuations than the retarded group. No significant overall change in EDA measures after ECT.	Cross-sectional
O’Kearney & Parry (Australia, 2014)	− 24 depressive episode (39.6±16.98 years; 67% females); − 24 PTSD (31.7±16.49 years; 50% females); − 24 healthy controls (35.8±15.73 years; 50% females).	SCR.	PTSD showed higher SCR during trauma recall compared with recall of other events and compared with depressed controls. No significant difference in reactivity between depressed and non-disordered participants.	Case-control (4)
Pazderka-Robinson et al. (2004)	− 43 patients with chronic fatigue syndrome (46.3±9.6 years; 100% females); − 25 depressed patients (35.3±10.5 years; 100% females); − 44 controls (27.8±9.3; 100% females).	SCL and SCR recorded during an orienting task.	SCLs were markedly lower for the chronic fatigue syndrome group, with no difference between controls and depressives.	Case-control (4)
Perez-Reyes & Cochrane (US, 1967)	− 108 neurotic depressed inpatients; − 82 psychotic depressed inpatients; − 69 healthy volunteers.	SCL and mean number of SCRs per minute.	No difference in initial SCL or SCR frequency, but significant differences in SCR susceptibility among the three groups.	Case-control (1)
Pruneti et al. (Italy, 2010)	− Outpatients (38.4±9.7 years; 52% females) with the following diagnoses: − Generalized Anxiety Disorder (GAD, n = 24); − Major Depression Episode (MDE, n = 14); − Panic Disorder (PAD, n = 12); − Obsessive-Compulsive Disorder (OCD, n = 10).	SCR registered in three consecutive phases: baseline (registration at rest), stress presentation, and recovery.	SCR mean values are much higher for GAD and PAD patients than for MDE and OCD. The amplitude of the SCR was also significantly different among groups.	Cross-sectional
Rohde et. al (Germany, 2014)	− 43 currently depressed patients (36.34±11.36 years; 60% female); − 36 controls (32.19±12.26 years; 66% female); − physiological data of 32 currently depressed participants and 23 controls.	SCR, EMG corrugator activity at baseline and during a Mindful Breathing Exercise. Mindful episode: the patient mindfully observed his/her breathing. Drifted episode: the patients had his/her mind wandering, not focused on the task.	SCR: no significant differences were found between currently depressed patients and controls. In both groups, greater SCRs recorded during drifted episodes, while weaker SCRs were elicited within mindful episodes.	Case-control (4)
Rottenberg et al. (2002, USA)	− 71 depressed persons (mean age 33.4 years; 66% females); − 33 non-depressed healthy controls (mean age 32.3 years; 70% females).	SCL and SCR recorded at baseline and after stimuli (neutral film and cry-inducing film).	During the sad film, non-depressed criers had greater number of SCR than non-depressed non-criers. Non-depressed non-criers exhibited significant decreases in SCL from the neutral to the sad film. Depressed criers exhibited significantly smaller increases in SCL and SCR rate to the sad film than did non-depressed criers.	Case-control (5)
Schneider (US, 1983)	− 10 depressed inpatients (9 with unipolar and 1 with bipolar disorder) (mean age 43.1 years; 0% females); − 23 schizophrenic inpatients (13 who underwent drug 'washout' and 10 who didn’t) (mean age 45.2 years; 0% females); − 10 healthy controls (mean age 40.9 years; 0% females).	SCL, SCR and NS.SCR recorded during exposure to tones.	SCLs were higher in the normal and schizophrenic groups than in the other two groups. The depressives' mean laterality index, unlike that of the controls, reflected lower SCLs and SCRs from the right hand than from the left. The indices from the other two patient groups did not significantly differ from controls’ ones. The patient groups generally had a lower proportion of 'responders' than did the health controls group, a lower number of SFs and a lower SF amplitude.	Case-control (2)
Schneider D. et al. (Germany, 2012)	− 28 in- and out-patients with unipolar MDD (age range 21–54 years; 43% females); − 28 age- and education-matched control participants.	Galvanic skin conductance (GSC), galvanic skin responses (GSR). Stimuli: 96 short video-clips conveying one of 4 emotion categories (happy, sad, fear, disgust) or no emotion (neutral).	Patients displayed more GSRs than healthy controls in all emotion categories. In patients, the number of GSRs did not differ between emotion categories, and neutral video clips evoked less GSRs than happy, sad, fearful and disgusted clips.	Case-control (4)
Siepmann et al. (Germany, 2001)	12 healthy volunteers (mean age 25 years; 0% females): − Reboxetine 4 mg, twice a day, for 13 days; − Placebo, twice a day, for 13 days.	SCL and SCR recorded following a single deep inspiration.	SCR was decreased after multiple dosing with reboxetine.	Crossover
Siepmann et al. (Germany, 2003)	12 healthy volunteers (mean age 34 years; 0% females): − Sertraline 50 mg, once a day, for 14 days; − Placebo, once a day, for 14 days.	SCL and SCR recorded following a single deep inspiration	Sertraline caused a significant reduction of SCL, whereas SCR was not changed.	Crossover
Siepmann et al. (Germany, 2004b)	12 healthy volunteers (mean age 27 years; 0% females): − Moclobemide 150 mg, twice a day, for 14 days; − Placebo, twice a day, for 14 days.	SCL and SCR recorded following a single deep inspiration	SCRs did not change after treatment with moclobemide.	Crossover
Siepmann et al. (Germany, 2004a)	12 healthy volunteers (25±3 years; 0% females): − Amitriptyline 25 mg, 3 times daily for 13 days; − St. John’s wort extract, 3 times daily for 13 days; − Placebo, 3 times daily for 13 days.	- SCR recorded following a single deep inspiration.	St. John’s wort extract had no effect on SCR. SCR significantly decreased during treatment with amitriptyline at all time points of measurement.	Crossover
Sigmon et al. (USA, 2007)	-15 MDD patients, Recurrent, with Seasonal Pattern (MDD-SAD; 80% females); -15 MDD patients (MDD; 66.7% females); -15 controls (80% females). Mean age 38.93 years.	Baseline SCL recordings obtained for a 5-min baseline period and average SCL collected across the baseline period. SCR at baseline and after presentation of winter and summer scenes were recorded.	MDD-SAD patients exhibited a greater number of SCR and greater SCR amplitude in reaction to the winter scenes, than individuals in the MDD and control groups. Individuals in the control and MDD groups did not significantly differ on frequency of SCR or strength of SCR amplitude in response to winter scenes. Individuals in the three groups did not significantly differ on SCR amplitude, in reaction to the summer scenes.	Case-control (5)
Silva et al. (Brazil, 2000)	-29 adult healthy volunteers (86% females) received - 100 mg Nefazodone (NF) (mean age 25 years); - 200 mg NF (mean age 26.6 years); - placebo (mean age 24.9 years). (Double-blind condition).	Test 1: (1) amplitude of SCR - fluctuations that occurred within 5 s from a sound stimulus; (2) number of spontaneous fluctuations – those occurring be yond the above mentioned 5-s window; (3) SCL – average level of conductance during the intervals between fluctuations.	Conditioned fear. NF decreased the number of spontaneous fluctuations of skin conductance in a dose-dependent way, although the drug did not affect the amplitude of the SCR to the tone.	Crossover Double-blind
Storrie et al. (US, 1981)	− 10 inpatients with Primary Affective Disorder, free from psychoactive medications for at least 3 days prior the initial testing (48±4 years; 0% females); − 10 healthy subjects without history of depressive dysfunction or psychosis (49±5 years; 0% females).	SCL and SCR recorded during Valsalva maneuvers. Measures repeated three weeks after initiation of therapy with tricyclic antidepressants or antipsychotic medication.	Laterality hypothesis not confirmed. Mean SCL and SCR significantly higher in the control group than in depressed patients. No significant difference in SCL and SCR between treated and untreated patients.	Case-control (5)
Thorell & D’Elia (Sweden, 1988)^3^	− 28 in- and out-patients with major depressive episode and dysthymic disorders (42.5; 50% females); − 59 mentally and somatically healthy subjects.	SCL at the onset of the first stimulus, mean SC fluctuation rate (SCFr) per minute, SCR magnitude (SCRm) to the first stimulus, the SCR rate (SCRr), and the index of SC nonresponding (SCRi).	Significantly higher EDA during remission than during depression according to all 5 EDA variables. No significant difference in EDA between patients in remission and healthy subjects. Among suicide attempters, EDA at follow-up did not change significantly, but was not significantly lower than in healthy subjects. However, SCRm and SCRi in suicide attempters were significantly lower at follow-up than in the healthy group. The extremely hyporesponsive patients, including suicide attempters, when in remission, did not reach the levels of the healthy subjects except for SCFr. The patient with recurrent major depressive episodes when in remission did not reach the electrodermal responsivity levels found in the healthy subjects.	Case-control (5)
Thorell et al. (1988, Sweden)	− 59 depressed patients; − 59 healthy controls.	After 5 min without stimulation, 37 tones were presented. SCL at onset of the first stimulus; stimulus-unrelated SC fluctuation rate (SCFr) during the whole stimulation session except for 10 s after onset of each stimulus; SC response magnitude (SCRm) to the first stimulus; SCR rate (SCRr); index of SC nonresponding (SCRi). Cortisol in plasma and in urine were dosed, dexamethasone suppression test (DST) was performed.	No significant correlation between EDA and cortisol in patients’ plasma and urine. No relationship between EDA and the outcome of DST, neither any simple relationship between EDA and cortisol in plasma and in urine. Positive correlation between SCL and morning plasma cortisol and negative correlations between EDR and nocturnal urinary cortisol in the suicide attempters. In the suicide attempters, the already extremely low EDR is additionally suppressed with increased nocturnal urinary cortisol excretion while SCL, and to some extent also EDR, are elevated with increased morning plasma cortisol. This pattern is opposite to that obtained in the healthy subjects and in the non-suicidal patients for SCL and plasma cortisol.	Case-control (5)
Thorell et al. (Sweden, 1993)	− 50 depressive patients (42.2±13.4 years; 52% females); − 50 age and gender-matched healthy subjects (42.6±13.5 years; 48% females).	Blood and urine for the measurement of basal hormone levels were collected at 8 a.m., immediately followed by clinical ratings and EDA measurements.	Positive relationships between SCL and basal levels of thyroid hormones in the healthy subjects were absent or reversed in the depressive patients. No relationships between electrodermal responsivity and thyroid hormones in depressive and healthy subjects. No significant difference between drug-free patients and patients treated with antidepressant medications regarding the EDA variables.	Case-control (4)
Toone et al. (UK, 1981)	− 22 schizophrenic patients (mean age 35.8 years; 64% females); − 11 unipolar depressed patients (mean age 40.6 years; 73% females); − 4 manic patients (mean age 38.7 years; 50% females); − 12 anxiety state patients (mean age 30.1 years; 50% females); − 22 healthy controls (mean age 36.7 in males and 39.3 in females; 36% females).	SCLs, SCRs and SF recorded at rest and during 32 flashes.	No group showed a distinctive pattern of lateral asymmetry; the only significant difference was in the adaptation of SCL during stimulation. Among depressed males the adaptation of SCL during stimulation on the left exceeded the right; among depressed females the trend was reversed. The frequency of SF in EDA was greater in the anxiety state and schizophrenic groups and in those patients who reported auditory hallucinations during recording.	Case-control (6)
Tsai et al. (US, 2003)	− 12 Spanish-speaking latinas with major depressive episode (28.28±7.45; 100% females); − 10 non-depressed Spanish-speaking latinas (28.28±7.45; 100% females).	SCL during sad and amusing film clips of human and animal content.	Depressed Latinas demonstrated less electrodermal reactivity across all the film clips than non-depressed Latinas.	Case-control (4)
Ward et al. (USA, 1983)	− 33 depressed patients (mean age 42.58 years; 36% females); − 71 healthy controls (mean age 35.59 years; 46% females).	SCL was examined during 15 minutes during the first week of hospitalization.	Healthy and depressed women had significantly lower SCLs than healthy and depressed men; subjects with recurrent depression had significantly lower SCLs than subjects with a first episode depression. Significant positive correlation between age and SCL in the depressed group but not in the control group. No significant SCL differences between medicated and unmedicated patients. No significant SCL differences between endogenous-nonendogenous, situational-non-situational, primary-secondary, or DST suppressor-DST non-suppressor depression. SCL proved a very sensitive and specific test for distinguishing non-geriatric unipolar depressed adults from physically healthy normal controls.	Case-control (3)
Ward & Doerr (1986)	− 37 in- and out- patients with major affective disorder (mean age 35.3 years; 59% females); − 71 controls (control group 1) (mean age 35.5 years; 46% females); − 334 "stressed" normal controls (control group 2) who were first parents of newborns (mean age 28.7 years; 51% females).	Resting SCL.	Lowest SCLs in the depression group. No difference between the two control groups. For women, SCL less than 3.0 micronho/cm2 yielded a sensitivity of 95% and a specificity of 91% for Control Group 1. For males, a criterion of SCL less than 4.8 yielded a sensitivity of 93% and a specificity of 89% for Control Group 1. No significant difference in SCL between drug-free and medicated patients. SCL was abnormally low in all depression subtypes.	Case-control (3)
Weckowicz et al (Alberta, 1971)	− 212 depressed inpatients included; data at follow-up available for 170 (mean age 38 years; 69% females).	Galvanic skin responses (GSR) to a noxious stimulus, obtained one minute after the last basal reading. Patients were tested after admission and before starting therapy. Second assessment after 3 weeks of therapy.	GSR was not a predictor for ECT, it was a near-significant predictor for psychotherapy and for drug therapy.	Cross-Sectional
Williams et al. (1985)	36 primary depressive patients: − 27 unipolar (37.4±12.5 years; 74% females); − 9 bipolar (46.2±10.1 years; 55% females).	SCL and SCR recorded for two experimental conditions: − auditory stimuli during relaxation; − a task requiring responding to specific tones pressing a foot pedal.	No differences in tonic or phasic EDA in unipolar or bipolar subtype, response to the dexamethasone suppression test, severity of depression, medication status, or gender. Patients with psychomotor retardation had significantly lower levels of tonic EDA than non-retarded ones.	Cross-sectional
Zullino et al. (Switzerland, 2015)	− 58 patients randomly assigned to 8-week Venlafaxine treatment (44.9±8 years; 36.7% females) or applied relaxation (45.6±11.3 years; 46.4% females).	Skin conductance.	Patients treated with Venlafaxine had significantly lower EDA than the other group, both at week 4 and 8.	Randomized comparative

Legend:

^1^The “Newcastle-Ottawa Scale (NOS) for assessing the quality of case-control and cohort studies” was used to evaluate the case-control (scores 0-10) and cohort studies (scores 0-12)

^2^Follow-up study of Iacono et al. (1983)

^3^Follow-up study of Thorell et al. 1987

CLBP= Chronic Low Back Pain; CS = Conditioned Stimuli; ECT=Electroconvulsive therapy; EDA = Electrodermal Activity; EDR = Electrodermal Response; GSC = Galvanic skin conductance; GSR = Galvanic skin response; MDD = Major Depressive Disorder; MDE = Major Depressive Episode; NS.SCR = Nonspecific Skin Conductance Response; OCD = Obsessive-Compulsive Disorder PTSD = Post-traumatic Stress Disorder; SAD = Seasonal Affective Disorder; SCL = Skin Conductance Level; SCFr = Skin Conductance Fluctuation rate; SCR = Skin Conductance Response; SF= Spontaneous Fluctuation; SR = Skin Resistance; SRR = Skin Resistance Response

### Association between EDA and depression

Although low EDA in depressed patients was first described in 1890 [32], interest in this physiological variable as a marker of depressive disorders occurred mainly between the late 1970s and the 1990s.

The studies included in this section are very varied, both regarding sample and EDA variables. For clarity they have been grouped under the following headings: Depressed patients vs. controls; Depression subgroups; Depressed patients vs. other psychiatric patients; Elicitation methods and EDA; Other studies of EDA characteristics; EDA and hormones; EDA and antidepressants.

#### Depressed patients vs. controls

Nine studies, with a NOS score ranging from 2 (2 studies) to 5 (3 studies), mean NOS score 3.8, reported lower SCL, increased SCR latency and lower SCR amplitude in depressed subjects compared with healthy controls [33–41]. Thorell et al. reported a lower level of EDA in depressed patients than in healthy subjects, reflected in lower central values for SCL, SCR amplitude, SCR rate and an index of non-response during neutral tone stimulation [42].

The overall efficiency of SCR as a means of discriminating between depressed and healthy subjects has been estimated to be about 80% [43]. It has also been reported that depressed patients show marginally faster habituation of the skin resistance orienting response than healthy controls [44, 45], although one study [35] with a lower NOS score than the others (2 vs. 3 and 5) did not find this.

Some researchers looked at age- and gender-related EDA variability. In adolescents [46] the results were consistent with the notion that EDA is lower in major depressive disorder (MDD) patients than in control subjects. In contrast SCL was similar in patients with dementia, depressed patients (mean age 75.9 ± 7 years) and healthy controls, probably due to a decline in the number of active sweat glands and sweat production in the elderly. As far as gender is concerned, SCLs were lower in women than in men in both depressed patients and healthy controls [47].

A study of GSRfound that patients were hyporesponsive because of depressive inhibition in an experimental condition, but failed to find any difference in GSR variables between patients and controls in two rest periods and in a no-response experimental condition [48].

Unlike most studies in this field Lapierre and Butter reported a higher SCL and higher basal skin resistance in depressed patients than in controls. The amplitude of SCR and number of non-specific SCRs were similar in depressed patients and controls [49]. Other studies reported higher SCLs in depressed subjects than in healthy controls [50, 51] and psychiatric controls [50]. A couple of studies [52, 53] failed to find any difference between depressed patients and healthy controls with respect to SCL [52] and SCR [53] variables.

It should be noted that the Lapierre and Butter study scored 0 on the NOS because of the lack of information about the study design, whereas the other studies [50–52] described above had higher NOS scores (5,4, and 4, respectively).

Briefly, lower EDA, especially lower SCL and SCR, in depressed patients than in healthy controls was the most consistently reported result.

#### Depression subgroups

It has been suggested that in depressed patients level of EDA may be a function of the type of depression. A recent, cross-sectional study offered preliminary evidence that EDA levels may differentiate the phases (depressive vs. mixed vs. euthymic) of bipolar disorder [54].

Lower EDA levels were found in patients with psychomotor retardation or symptoms of inhibition than in agitated depressed patients ([55, 56, 58], mean NOS score 4.3; [57, 59] cross-sectional studies), except for the study by Lapierre and Butter, which was the least robust in terms of NOS score [49; NOS score 0]. Patients classified as suffering from “psychotic” (rather than “neurotic”) [60] and “endogenous” (rather than “non-endogenous”) [61, 62] depression had lower EDA levels, although this finding was not consistent across all studies. Moreover, the published research on this issue [40, 58, 59, 63] is highly heterogeneous with respect to NOS score (2, 5, not applicable, and 1, respectively).

The absence of a SCR was observed in depressed patients with predominance of anxiety symptoms [40].

In brief, patients classified as suffering from “psychotic” and “endogenous” depression had lower EDA levels compared to “neurotic” and “non-endogenous” depression. However, in view of the significant differences in the methodology and design of the relevant studies and their inconsistent results, the utility of EDA as a means of discrimination between subgroups of depressive patients remains to be demonstrated.

#### Depressed patients vs. other psychiatric patients

Comparisons of EDA in depressed patients and patients with other psychiatric disorders have been performed to ascertain whether EDA can be used as a marker of depressive states.

EDA abnormalities have been described in schizophrenic patients [64]. Compared with normal controls, both schizophrenic and depressed patients showed high levels of non-response in the habituation series, but schizophrenics - unlike the depressed patients - showed a decrease in SCR non-response to the target tone [55, 65]. In contrast Levinson [51] found no substantial differences between schizophrenic and depressed patients and normal controls with respect to SCR.

Pruneti et al. [66] found that patients with generalised anxiety disorder or panic disorder had higher SCRs than patients with major depression or obsessive-compulsive disorder.

Have et al. [67] reported that depressed patients, patients with degenerative dementia of the Alzheimer type and healthy controls had similar SCLs.

In summary, there have been only six studies comparing depressed patients to other psychiatric patients, and they deal with different disorders, making it hard to draw clear conclusions.

#### Elicitation methods and EDA

The published research using emotional elicitation protocols and other tasks to investigate EDA is highly heterogeneous with respect to sample, EDA variables, task and NOS score (range: 3 - 6), making it very hard to compare studies and to draw unequivocal conclusions.

Lower EDA, lower SCRs and higher SCL/SCRs were found in depressed subjects compared with controls in response to various emotional elicitation protocols (including exposure to pleasant and unpleasant pictures, exposure to sad and amusing film clips, and suppression of emotional reaction to pictures) [68–71]. Other studies found contrasting or mixed results depending on the type of task [72–75, 50]. For instance, in a standardised mood induction experiment, MDD patients had higher SCRs than controls in the cartoon condition, but not when mood was induced through happy and neutral pictures [73]. Schneider et al. [75] found that depressed subjects showed increased reactivity and autonomic arousal (elevated GSRs) in response to affective stimuli, compared to healthy controls [75]. Rohde and coworkers [76] studied depressed patients and healthy controls performing a Mindful Breathing Exercise task and found no difference in SCR between the two groups.

A couple of studies including elicitation protocols compared depressed patients with a seasonal pattern (seasonal affective disorder) and healthy controls. When exposed to overcast stimuli [77] or winter scenes [78] patients with seasonal affective disorder displayed more frequent SCRs and SCRs of greater magnitude, whereas the opposite pattern was found for sunny stimuli [77] and there was no disease by stimulus interaction for SCLs [77].

In short, the heterogeneity of study designs does not allow to draw clear conclusions in this field.

#### EDA and hormones

Only two studies dealt with this topic and they concerned different hormones, so it is not possible to generalise from the results. Based on the dexamethasone suppression test EDA in depressive patients does not appear to be related to dysfunctions of the hypothalamic-pituitary-adrenal axis. On the other hand, suicide attempters exhibited opposite correlations between EDA and cortisol in plasma and in urine, suggesting that there may be a complex relationship between EDA and cortisol production [79].

The positive correlations found between basal levels of thyroid hormones and SCL in healthy subjects were absent or reversed in depressed patients [80].

#### EDA and antidepressants

A cross-sectional study by Weckowicz [81] found GSR was a near-significant predictor of psychotherapy and drug therapy in depressed patients.

Several studies [37, 43, 55, 59, 82] failed to find any difference in EDA levels in depressed patients in response to antidepressant treatment or other medication, but it is hard to draw clear conclusions from these studies, many of which assessed non-specified antidepressants and/or antipsychotic medication.

Other studies investigating the effects of antidepressant compounds on EDA have found different results, but they cover a range of drugs and the results are mixed. Of the studies selected for this review, 6 assessed the effects of tricyclic antidepressants (imipramine [83, 84], amitriptyline [85–88]); 1 a tetracyclic antidepressant (maprotiline; [83]); 1 a serotonin antagonist and reuptake inhibitor (nefazodone; [89]); 3 selective serotonin reuptake inhibitors (paroxetine; [84], sertraline; [90], fluoxetine; [91]); 1 a noradrenaline reuptake inhibitor (reboxetine; [92]); 1 a serotonin-noradrenaline reuptake inhibitor (venlafaxine; [93]); 1 a reversible inhibitor of monoamine oxidase A (moclobemide; [94]); and 1 an unspecified antidepressant [95].

Imipramine-treated patients showed lower EDA than controls [84]. Regarding amitriptyline, findings were not consistent across studies, spanning from no correlation between the drug plasma level and EDA measures [86], to lower activation and decreased NS.SCRs and SCR in patients treated with amitriptyline [78, 87, 88]. Reboxetine reduced SCR after multiple dosing [92], while sertraline, moclobemide and nefazodone-treated patients showed no change in SCR [90, 94, 89]. On the other hand, sertraline-treated patients had lower SCL compared to controls [90]. Venlafaxine caused a reduction of EDA measures [93]. See Tables 1 and 2 for further details.

Very briefly, studies of EDA and antidepressants, either found no correlation between drugs and EDA measures, or a reduction of EDA measures (NS.SCRs, SCR, SCL) in subjects taking medications.

#### Other studies of EDA characteristics

A couple of follow-up studies investigated the temporal stability of EDA. In the studies of Iacono and coworkers [42, 45] the EDA variables were moderately stable at the one-year follow-up, whereas in the study of Thorell and d’Elia [43, 61] patients’ EDA was elevated in the remission phase and similar to that of the matched healthy subjects. It should be noted, however, that the mean follow-up period in this study was 2 years, which suggests that the recovery of tonic EDA in patients with affective disorders is probably a very slow process. Thorell and d’Elia’s study had a more robust design than the Iacono studies (NOS 5 vs. NOS 3).

A one-year cross-sectional follow-up study of depressive and depressive-anxious patients found mixed results with respect to the temporal stability of EDA: changes were smallest in stable patients, and greatest in all four labile-activated patients [85].

The laterality of EDA has also been explored and it has been hypothesised that there is right-hemisphere hyperexcitability in depressive conditions. After the original 1978 study by Myslobodsky and Horesh [96] others reported that EDA levels were lower on the right hand than the left under various experimental conditions, including rest [97]. Nevertheless, contrasting results were obtained both at rest and during stimulation [65, 98], and during different phases of illness, including remission [37, 99]. Notably, the first two studies cited in this paragraph had lower NOS scores (3 and 2) than the last four (respectively 4, 6, 5, 5); the existence of lateral differences in EDA remains to be confirmed.

### EDA and suicidal behaviour

It has been suggested that differences in EDA may be specific to suicidality rather than depression [100]. Although there has been less research on EDA in individuals exhibiting suicidal behaviour than in people with depression, there is consistent evidence of electrodermal hypoactivity in depressed suicide attempters compared with non-suicidal depressed patients and healthy controls [61, 58, 101, 102].

Correlations between EDA and the type and level of suicide risk have been suggested. For instance, one study compared patients recently admitted to the hospital because of suicide threats or preoccupations, but with no history of attempts, controls with no history of suicide threats or attempts and no reported suicidal thoughts at the time of data collection and suicide attempters with a history of one or more suicide attempts. The first group showed the smallest GSR to the word ‘suicide’, suggesting generally lower reactivity [103].

Other studies have assessed SCR habituation in individuals with different patterns of suicidal behaviour. Violent suicide attempters and suicide completers were both found to be fast habituators [101, 104, 105]. The findings on non-violent suicide attempters, patients with suicidal ideation and non-suicidal depressed patients are less clear: one study found that non-violent suicide attempters showed either fast or slow habituation [101] but another found no differences among these groups (violent and non-violent suicide attempters, patients with suicidal ideation, non suicidal patients) [104]. Jandl et al. found no difference in the habituation of violent and non-violent suicide attempters, but corroborated the general finding of hyporeactivity in suicide attempters compared with non-attempters [106]. A study comparing parasuicidal adolescent girls with healthy controls found no differences in EDA variables [107].

Thorell et al. [24] carried out a meta-analysis of earlier research covering a total of 297 depressed patients and 59 healthy subjects. Electrodermal hyporeactivity was strongly associated with high suicide risk. Extremely low electrodermal reactivity had a sensitivity of 96.6% and a specificity of 92.9% for suicide and a sensitivity of 83.3% and specificity of 92.7% for suicide and/or violent suicide attempt.

A further analysis of data from 783 depressive patients by Thorell et al. [108] confirmed that electrodermal hyporeactivity is a marker of suicidal tendency in both unipolar and bipolar depression, independently of severity of depression, trait anxiety, gender and age.

Recently the protocol for a study involving 1573 patients with a primary diagnosis of depression recruited from 15 centres in nine European countries that will test the predictive value of electrodermal hyporeactivity (measured with the electrodermal orienting reactivity EDOR test) for suicide and suicide attempt has been published. The extant literature suggests that suicide attempt with intent to die and completed suicide will be associated with electrodermal hyporeactivity [27].

## Discussion

The aim of this review is to offer a comprehensive overview of the EDA literature with a view to assessing its potential utility as a biomarker of depressive states and risk of suicidal behaviour, and its potential role in advanced, integrated physiological evaluation systems.

Overall, our review of the literature supports the hypothesis that electrodermal hypoactivity is a feature of depression. Nevertheless, considering the number and robustness of studies, EDA seems to be more useful in discriminating depressive patients from healthy controls than from other psychiatric patients. Moreover, specific EDA features (e.g., SCL, SCR, NS.SCR, habituation rate, etc.) seem to be differently affected, and the extremely contrasting nature of studies and of the variables used cannot be overlooked. Briefly, specific patterns of electrodermal hypoactivity may be a reliable marker of a depressive state at population level, but they should be carefully combined with other physiological and non-physiological indicators when used for preventive and diagnostic purposes.

Another area that deserves further investigation is the potential use of EDA to distinguish between subtypes of depression. At present the evidence suggests that patients with psychomotor retardation, endogenous and psychotic depression show lower EDA values than patients with agitation, non-endogenous and neurotic depression, respectively.

It has been hypothesised that electrodermal hypoactivity is a rather stable trait of patients affected by depression, although increases in EDA may indicate euthymia or remission [61, 54]. It should be noted that extremely hyporesponsive depressive patients, including suicide attempters and patients with recurrent major depression, may fail to reach the EDA levels of healthy subjects even when in remission [54].

There is even more debate about the effects of antidepressants on EDA and research has yielded mixed results. In the context of experimental anxiety conditioning tasks some antidepressants blunt EDA in healthy subjects, but the data from depressed patients are not consistent and there is no clear correlation between EDA and clinical improvement. As Thorell hypothesised, electrodermal hypoactivity may be a rather stable trait of depressed patients; EDA appears to be only marginally affected by treatment and clinical improvement, and normalisation may not occur until several months - or even years - after clinical recovery [61]. Moreover, there is only limited evidence in relation to each drug and for most drugs it comes from just one study.

As far as suicidal behaviour is concerned, extreme hyporeactivity has been consistently reported in both suicide attempters and at baseline in subjects who eventually committed suicide during a follow-up period; moreover, hyporeactivity seems to be related to the choice of a violent method for attempted or completed suicide [101, 104, 105]. On this basis it has been hypothesised that extreme electrodermal hypoactivity is a marker of suicidal tendencies in depressed patients, and it appears to be independent of severity of depression [61, 102]. Recent studies [24, 108] showed that EDA discriminates well between patients who will subsequently commit suicide, make a non-violent suicide attempt or make a violent suicide attempt, but it is less clear that EDA can be used to distinguish individuals with current suicidal ideation from depressed patients who are not currently suicidal. Nevertheless, it has been suggested that the evidence is sufficient to warrant strict monitoring of both euthymic and depressed patients who are show extreme electrodermal hyporeactivity, even in the absence of suicidal ideation. Obviously close monitoring and adequate antidepressant therapy are even more necessary in hyporeactive patients with suicidal ideation [24].

### Assessment of the robustness of the synthesis and limitations

We adhered to the PRISMA statement [28, 29], which requires the use of defined inclusion/exclusion criteria, a rigorous search strategy and assessment of the quality assessment of included studies. Nevertheless, several limitations of this review should be acknowledged. First, as shown by the NOS scores, the quality of several of the included studies is questionable and most are quite old. The criteria for ‘depression’ vary somewhat between studies. Most of studies used the Diagnostic and Statistical Manual of Mental Disorders (DSM-III and DSM-IV) criteria [109, 110], but some used the International Classification of Diseases (ICD-9 and ICD-10) [111, 112], the Research Diagnostic Criteria [113], the Feighner Research Criteria or multiple classification systems [114]. Definitional inconsistency is also a problem in comparisons of subtypes of depression as there are no standard criteria for distinguishing between, for example, retarded and agitated depression, endogenous and non-endogenous depression or psychotic and neurotic depression. Furthermore, most studies included patients with various mood disorder diagnoses (e.g., unipolar depression, bipolar depression, dysthymia), without specifying the illness phase or the treatment patients were receiving. Finally, comorbidity was not always accounted for in comparisons between depression and other pathological conditions.

Besides diagnostic issues, over time the EDA assessment methods have changed, leading to inconsistencies. Not all the studies have assessed the same EDA parameters, and despite improvements in measuring equipment since the discovery of electrodermal phenomena more than 100 years ago [32], much of the research reviewed in the present article is limited to observational measurements performed over short periods of time, in laboratory settings or artificial clinical environments.

## Conclusions

This review offers a more comprehensive assessment of the extant EDA literature than previous ones [25, 26] and it corroborates their findings, namely that there are associations between electrodermal hypoactivity and depression and suicidal behaviour. EDA appears to be a reliable marker, with high sensitivity and specificity, of depressive states, suicidal tendencies and suicidal behaviour [24, 39, 43, 108]. Nevertheless, further studies are required to validate EDA as an indicator of other clinical features, such as depression subtypes, response to treatment and acute suicide risk.

## Appendix 1

**Table 3:** Search strings

Search Engine	Search String
PubMed (advanced search, All fields)	*((electrodermal activity) OR (electrodermal response) OR (skin conductance response) OR (skin conductance level)) AND ((depression) OR (depressive disorder) OR (mood disorder) OR (affective disorder) OR (suicide) OR (suicide attempt) OR (suicidal behavior) OR (suicidal ideation) OR (antidepressant))*
Scopus	*((TITLE-ABS-KEY ((“electrodermal activity” OR “electrodermal response” OR “skin conductance response” OR “skin conductance level”)) AND TITLE-ABS-KEY ((“depression” OR “depressive disorder” OR “mood disorder” OR “affective disorder” OR “suicide” OR “suicide attempt” OR “suicidal behavior” OR “suicidal ideation” OR “antidepressant”)))*
Cochrane Library	(“electrodermal activity” OR “electrodermal response” OR “skin conductance response” OR “skin conductance level”) AND (“depression” OR “depressive disorder” OR “mood disorder” OR “affective disorder” OR “suicide” OR “suicide attempt” OR “suicidal behavior” OR “suicidal ideation” OR “antidepressant”)
Web of Science	*((TITLE-ABS-KEY ((“electrodermal activity” OR “electrodermal response” OR “skin conductance response” OR “skin conductance level”)) AND TITLE-ABS-KEY ((“depression” OR “depressive disorder” OR “mood disorder” OR “affective disorder” OR “suicide” OR “suicide attempt” OR “suicidal behavior” OR “suicidal ideation” OR “antidepressant”)))*

## Abbreviations

EDA: Electrodermal activity
GSR: Galvanic skin response
MDD: Major depressive disorder
NS.SCR: Non-specific skin conductance response
PRISMA: Preferred reporting items for systematic reviews and meta-analyses
SCL: Skin conductance level
SCR: Skin conductance response
SRR: Skin resistance response

## References

[1] Sudol K, Mann JJ (2017). Biomarkers of suicide attempt behavior: towards a biological model of risk. Curr Psychiatry Rep.

[2] Öhman A, Hamm A, Hugdahl K. Cognition and the autonomic nervous system: orienting, anticipation, and conditioning. In Cacioppo JT, Tassinary LG & Berntson GG (Eds.), Handbook of psychophysiology (2nd ed.). New York, NY: US: Cambridge University Press; 2000. pp. 533–575.

[3] Pandey GN, Dwivedy Y. The neurobiological basis of suicide. Boca Raton (FL): CRC Press, Taylor & Francis, Chapter 20 Peripheral biomarkers for suicide, 2012.

[4] Groscurth P (2002). Anatomy of sweat glands. Curr Probl Dermatol.

[5] Gunnar Wallin B, Fagius J (1986). The sympathetic nervous system in man — aspects derived from microelectrode recordings. Trends Neurosci.

[6] Asahina M, Suzuki A, Mori M, Kanesaka T, Hattori T (2003). Emotional sweating response in a patient with bilateral amygdala damage. Int J Psychophysiol.

[7] Bradley MM, Lang PJ. Measuring emotion: behavior, feeling and physiology. In Cognitive Neuroscience of Emotion. Eds. R.D. Lane and L. Nadel. New York: Oxford University Press. 2000.

[8] Boucsein, W. Electrodermal activity (2nd ed.). New York: Springer Science & Business Media; 2012.

[9] Braithwaite JJ, Watson DG, Jones R, Rowe M (2013). A guide for analysing electrodermal activity (EDA) & skin conductance responses (SCRs) for psychological experiments. Psychophysiology.

[10] Yoon JH, Ko CM, Ahn YS, Park KS, Choe KH, Yoo KJ (1994). Mechanism of decrease in heart rate by peripheral dopaminergic D2-receptors. Yonsei Med J.

[11] Lee GP, Arena JG, Meador KJ, Smith JR, Loring DW, Flanigin HF (1988). Changes in autonomic responsiveness following bilateral amygdalectomy in humans. Neuropsychiatry, Neuropsychol Behav Neurol.

[12] Mangina CA, Beuzeron-Mangina JH (1996). Direct electrical stimulation of specific human brain structures and bilateral electrodermal activity. Int J Psychophysiol.

[13] Lang PJ, Greenwald MK, Bradley MM, Hamm AO (1993). Looking at pictures: affective, facial, visceral, and behavioral reactions. Psychophysiology.

[14] Manning SK, Melchiori MP (1974). Words that upset Urban College students: measured with GSRs and rating scales. J Soc Psychol.

[15] Winton WM, Putnam LE, Krauss RM (1984). Facial and autonomic manifestations of the dimensional structure of emotion. J Exp Soc Psychol.

[16] Lacey JI, Lacey BC (1958). The relationship of resting autonomic activity to motor impulsivity. Res Publ Assoc Res Nerv Ment Dis.

[17] Mundy-Castle AC, McKiever BL (1953). The psychophysiological significance of the galvanic skin response. J Exp Psychol.

[18] Crider A, Kremen WS, Xian H, Jacobson KC, Waterman B, Eisen SA (2004). Stability, consistency, and heritability of electrodermal response lability in middle-aged male twins. Psychophysiology.

[19] Gottesman II, Hanson DR (2005). Human development: biological and genetic processes. Annu Rev Psychol.

[20] Iacono WG (1998). Identifying psychophysiological risk for psychopathology: examples from substance abuse and schizophrenia research. Psychophysiology.

[21] Crider A. Electrodermal response Lability-stability: individual difference correlates. In J.-C. Roy, W. Boucsein, D. C. Fowles, & J. H. Gruzelier (Eds.), Progress in Electrodermal Research Springer US; 1993. New York: Plenum. 1993:173–186.

[22] Crider A (2008). Personality and Electrodermal response Lability: an interpretation. Applied Psychophysiology and Biofeedback.

[23] Straub R, Jandl M, Wolfersdorf M (2003). Depressive state and Electrodermal activity of depressed inpatients during an acute suicidal state. Psychiatr Prax.

[24] Thorell LH (2009). Valid electrodermal hyporeactivity for depressive suicidal propensity offers links to cognitive theory. Acta Psychiatr Scand.

[25] Straub R, Hole G, Wolfersdorf M (1992). Electrodermal hypoactivity in depression: psychobiological marker or differential psychophysiologic disposition?. Schweiz Arch Neurol Und Psychiatrie (1985).

[26] Vahey, R., Becerra, R. (2015). Galvanic Skin Response in Mood Disorders: A Critical Review. 2015. http://www.redalyc.org/articulo.oa?id=56041176008. Accessed 15 Dec 2016.

[27] Sarchiapone M, Iosue M, Carli V, Amore M, Baca Garcia E, Batra A (2017). EUDORA multicentre research program: a naturalistic, European multicentre clinical study of EDOR test in adult patients with primary depression. BMC Psychiatry.

[28] Moher D, Shamseer L, Clarke M, Ghersi D, Liberati A, Petticrew M (2015). Preferred reporting items for systematic review and meta-analysis protocols (PRISMA-P) 2015 statement. Syst Rev.

[29] Shamseer L, Moher D, Clarke M, Ghersi D, Liberati A, Petticrew M (2015). Preferred reporting items for systematic review and meta-analysis protocols (PRISMA-P) 2015: elaboration and explanation. The BMJ.

[30] Popay J, Roberts H, Sowden A, Petticrew M, Arai L, Rodgers M, et al. Guidance on the conduct of narrative synthesis in systematic reviews. A Product from the ESRC Methods Programme. Version, 1. 2006 http://www.lancaster.ac.uk/shm/research/nssr/research/dissemination/publications/NS_Synthesis_Guidance_v1.pdf. Accessed 15 Dec 2016.

[31] Wells GA, Shea B, O’connell D, Peterson JEA, Welch V, Losos M, et al. The Newcastle-Ottawa scale (NOS) for assessing the quality of nonrandomised studies in meta-analyses. 2000. http://www.medicine.mcgill.ca/rtamblyn/Readings/The%20Newcastle%20-%20Scale%20for%20assessing%20the%20quality%20of%20nonrandomised%20studies%20in%20meta-analyses.pdf. Accessed 15 Dec 2016.

[32] Vigouroux A (1890). Etude sur la résistance électrique chez les mélancoliques.

[33] Carney RM, Hong BA, Kulkarni S, Kapila A (1981). A comparison of EMG and SCL in normal and depressed subjects. Pavlov J Biol Scie.

[34] Dawson ME, Schell AM, Catania JJ (1977). Autonomic correlates of depression and clinical improvement following electroconvulsive shock therapy. Psychophysiology.

[35] Miquel M, Fuentes I, Garcia-Merita M, Rojo L (1999). Habituation and sensitization processes in depressive disorders. Psychopathology.

[36] Storrie MC, Doerr HO, Johnson MH (1981). Skin conductance characteristics of depressed subjects before and after therapeutic intervention. J Nerv.

[37] Bonnet A, Naveteur J (2004). Electrodermal activity in low back pain patients with and without comorbid depression. Int J Psychophysiol.

[38] Donat DC, McCullough JP (1983). Psychophysiological discriminants of depression at rest and in response to stress. J Clin Psychol.

[39] Ward NG, Doerr HO (1986). Skin conductance: a potentially sensitive and specific marker for depression. J Nerv Ment Dis.

[40] Kamenskaya VM, Mikhailova FS (1985). Ratios of electroencephalographic and autonomic indexes in a stress situation in patients with different types of depression. Neurosci Behav Physiol.

[41] Iacono WG, Lykken DT, Peloquin LJ, Lumry AE, Valentine RH, Tuason VB (1983). Electrodermal activity in euthymic unipolar and bipolar affective disorders. A possible marker for depression. Arch Gen Psychiatry.

[42] Thorell LH, Kjellman BF, d’Elia G (1987). Electrodermal activity in antidepressant medicated and unmedicated depressive patients and in matched healthy subjects. Acta Psychiatr Scand.

[43] Dawson ME, Schell AM, Braaten JR, Catania JJ (1985). Diagnostic utility of autonomic measures for major depressive disorders. Psychiatry Res.

[44] Iacono WG, Peloquin LJ, Lykken DT, Haroian KP, Valentine RH, Tuason VB (1984). Electrodermal activity in euthymic patients with affective disorders: one-year retest stability and the effects of stimulus intensity and significance. J Abnorm Psychol.

[45] Giedke H, Heimann H (1987). Psychophysiological aspects of depressive syndromes. Pharmacopsychiatry.

[46] Mestanikova A, Ondrejka I, Mestanik M, Hrtanek I, Snircova E, Tonhajzerova I (2016). Electrodermal activity in adolescent depression. Adv Exp Med Biol.

[47] Ward NG, Doerr HO, Storrie MC (1983). Skin conductance: a potentially sensitive test for depression. Psychiatry Res.

[48] Giedke H, Bolz J, Heimann H (1980). Evoked potentials, expectancy wave, and skin resistance in depressed patients and healthy controls. Pharmakopsychiatr Neuropsychopharmakol.

[49] Lapierre YD, Butter HJ (1980). Agitated and retarded depression. Neuropsychobiology.

[50] Lewinsohn PM, Lobitz WC, Wilson S (1973). “Sensitivity” of depressed individuals to aversive stimuli. J Abnorm Psychol.

[51] Levinson DF (1991). Skin conductance orienting response in unmedicated RDC schizophrenic, schizoaffective, depressed, and control subjects. Biol Psychiatry.

[52] Pazderka-Robinson H, Morrison JW, Flor-Henry P (2004). Electrodermal dissociation of chronic fatigue and depression: evidence for distinct physiological mechanisms. Int J Psychophysiol.

[53] O'Kearney R, Parry L (2014). Comparative physiological reactivity during script-driven recall in depression and posttraumatic stress disorder. J Abnorm Psychol.

[54] Greco A, Valenza G, Lanata A, Rota G, Scilingo EP (2014). Electrodermal activity in bipolar patients during affective elicitation. IEEE J Biomed Health Inform.

[55] Bernstein AS, Schnur DB, Bernstein P, Yeager A, Wrable J, Smith S (1995). Differing patterns of electrodermal and finger pulse responsivity in schizophrenia and depression. Psychol Med.

[56] Lader MH, Wing L (1969). Physiological measures in agitated and retarded depressed patients. J Psychiatr Res.

[57] Noble P, Lader M (1971). The symptomatic correlates of the skin conductance changes in depression. J Psychiatr Res.

[58] Thorell LH, Kjellman BF, d’Elia G (1987). Electrodermal activity in relation to diagnostic subgroups and symptoms of depressive patients. Acta Psychiatr Scand.

[59] Williams KM, Iacono WG, Remick RA (1985). Electrodermal activity among subtypes of depression. Biol Psychiatry.

[60] Byrne DG (1975). A psychophysiological distinction between types of depressive states. Aust N Z J Psychiatry.

[61] Thorell LH, d’Elia G (1988). Electrodermal activity in depressive patients in remission and in matched healthy subjects. Acta Psychiatr Scand.

[62] Mirkin AM, Coppen A (1980). Electrodermal activity in depression: clinical and biochemical correlates. Br J Psychiatry.

[63] Perez-Reyes M, Cochrane C (1967). Differences in sodium thiopental susceptibility of depressed patients as evidenced by the galvanic skin reflex inhibition threshold. J Psychiatr Res.

[64] Dawson ME, Schell AM (2002). What does electrodermal activity tell us about prognosis in the schizophrenia spectrum?. Schizophr Res.

[65] Bernstein AS, Riedel JA, Graae F, Seidman D, Steele H, Connolly J (1988). Schizophrenia is associated with altered orienting activity: depression with electrodermal (cholinergic?) deficit and normal orienting response. J Abnorm Psychol.

[66] Pruneti CA, Lento RM, Fante C, Carrozzo E, Fontana F (2010). Autonomic arousal and differential diagnosis in clinical psychology and psychopathology. J Psychopathology.

[67] Have G, Kolbeinsson H, Pétursson H (1991). Dementia and depression in old age: psychophysiological aspects. Acta Psychiatr Scand.

[68] Mardaga S, Hansenne M (2009). Autonomic aspect of emotional response in depressed patients: relationships with personality. Neurophysiol Clin.

[69] Lemaire M, El-Hage W, Frangou S (2015). Increased affective reactivity to neutral stimuli and decreased maintenance of affective responses in bipolar disorder. European Psychiatry.

[70] Tsai JL, Pole N, Levenson RW, Muñoz RF (2003). The effects of depression on the emotional responses of Spanish-speaking Latinas. Cult Divers Ethn Minor Psychol.

[71] Rottenberg J, Gross JJ, Wilhelm FH, Najmi S, Gotlib IH (2002). Crying threshold and intensity in major depressive disorder. J Abnorm Psychol.

[72] Branković SB (2008). System identification of skin conductance response in depression–an attempt to probe the neurochemistry of limbic system. Psychiatr Danub.

[73] Falkenberg I, Kohn N, Schoepker R, Habel U. (2012). Mood induction in depressive patients: a comparative multidimensional approach. PLoS ONE. 2012; 7(1) e30016; doi:10.1371/journal.pone.0030016. 10.1371/journal.pone.0030016 PMC3253810 22253861

[74] Nissen C, Holz J, Blechert J, Feige B, Riemann D, Voderholzer U (2010). Learning as a model for neural plasticity in major depression. Biol Psychiatry.

[75] Schneider D, Regenbogen C, Kellermann T, Finkelmeyer A, Kohn N, Derntl B (2012). Empathic behavioral and physiological responses to dynamic stimuli in depression. Psychiatry Res.

[76] Rohde K, Adolph D, Dietrich DE, Michalak J (2014). Mindful attention regulation and non-judgmental orientation in depression: a multi-method approach. Biol Psychol.

[77] Lindsey KT, Rohan KJ, Roecklein KA, Mahon JN (2011). Surface facial electromyography, skin conductance, and self-reported emotional responses to light- and season-relevant stimuli in seasonal affective disorder. J Affect Disord.

[78] Sigmon ST, Whitcomb-Smith S, Boulard NE, Pells JJ, Hermann BA, Edenfield TM (2007). Seasonal reactivity: Attentional bias and Psychophysiological arousal in seasonal and nonseasonal depression. Cogn Ther Res.

[79] Thorell LH, Kjellman BF, d’Elia G, Kagedal B (1988). Electrodermal activity in relation to cortisol dysregulation in depressive patients. Acta Psychiatr Scand.

[80] Thorell LH, Kjellman BF, d’Elia G (1993). Electrodermal activity in relation to basal and post-dexamethasone of thyroid stimulating hormone and basal levels of thyroid hormones in major depressive patients and healthy subjects. Psychiatry Res.

[81] Weckowicz TE, Yonge KA, Cropley AJ, Muir W (1971). Objective therapy predictors in depression: a multivariate approach. J Clin Psychol.

[82] Bob P, Jasova D, Raboch J (2011). Subclinical Epileptiform process in patients with Unipolar depression and its indirect Psychophysiological manifestations. PLoS One.

[83] Lapierre YD, Butter HI (1978). Imipramine and maprotiline in agitated and retarded depression: a controlled psychiatric and psychiphysical assessment. Prog Neuro-Psychopharmacology.

[84] Barg T, Wolfersdorf M, Ruppe A (1996). The influence of various antidepressants on heart rate and Electrodermal activity during Psychophysiological examinations. Pharmacopsychiar.

[85] Heimann H (1978). Changes of psychophysiological reactivity in affective disorders. Arch Psychiatr Nervenkr.

[86] Breyer-Pfaff U, Gaertner HJ, Giedke H (1982). Plasma levels, psychophysiological variables, and clinical response to amitriptyline. Psychiatry Res.

[87] Ikeda Y, Nomura S, Sawa Y, Nakazawa T (1982). The effects of antidepressants on the autonomic nervous system-a current investigation. J Neural Transm.

[88] Siepmann M, Kirch W, Krause S, Joraschky P, Mueck-Weymann M (2004). The effects of St. John’s wort extract and amitriptyline on autonomic responses of blood vessels and sweat glands in healthy volunteers. J Clin Psychopharmacol.

[89] Silva M, Hetem LAB, Guimaraes FS, Graeff FG (2001). Opposite effects of nefazodone in two human models of anxiety. Psychopharmacology.

[90] Siepmann M, Grossmann J, Mück-Weymann M, Kirch W (2003). Effects of sertraline on autonomic and cognitive functions in healthy volunteers. Psychopharmacology.

[91] Fraguas R, Marci C, Fava M, Iosifescu DV, Bankier B, Loh R (2007). Autonomic reactivity to induced emotion as potential predictor of response to antidepressant treatment. Psychiatry Res.

[92] Siepmann M, Mück-Weymann M, Joraschky P, Kirch W (2001). The effects of reboxetine on autonomic and cognitive functions in healthy volunteers. Psychopharmacology.

[93] Zullino D, Chatton A, Fresard E, Stankovic M, Bondolfi G, Borgeat F (2015). Venlafaxine versus applied relaxation for generalized anxiety disorder: a randomized controlled study on clinical and electrophysiological outcomes. Psychiatr Q.

[94] Siepmann M, Handel J, Mueck-Weymann M, Kirch W (2004). The effects of moclobemide on autonomic and cognitive functions in healthy volunteers. Pharmacopsychiatry.

[95] Hattangadi S, Lidsky A, Lee H, Ban TA (1968). Orienting-reflex behavior and clinical psychopathology. Cond reflex.

[96] Myslobodsky MS, Horesh N (1978). Bilateral electrodermal activity depressive patients. Biol Psychol.

[97] Schneider SJ (1983). (1983). Multiple measures of hemispheric dysfunction in schizophrenia and depression. Psychol Med.

[98] Toone BK, Cooke E, Lader MH (1981). Electrodermal activity in the affective disorders and schizophrenia. Psychol Med.

[99] Iacono WG, Tuason VB (1983). Bilateral electrodermal asymmetry in euthymic patients with unipolar and bipolar affective disorders. Biol Psychiatry.

[100] Wolfersdorf M, Straub R, Barg T (1996). Electrodermal activity (EDA) and suicidal behavior. Crisis.

[101] Edman G, Åsberg M, Levander S, Schalling D (1986). Skin conductance habituation and cerebrospinal fluid 5-hydroxyindoleacetic acid in suicidal patients. Arch Gen Psychiatry.

[102] Thorell LH (1987). Electrodermal activity in suicidal and nonsuicidal depressive patients and in matched healthy subjects. Acta Psychiatr Scand.

[103] Spiegel D (1969). Autonomic reactivity in relation to the affective meaning of suicide. J Clin Psychol.

[104] Keller F, Wolfersdorf M, Straub R, Hole G (1991). Suicidal behaviour and electrodermal activity in depressive inpatients. Acta Psychiatr Scand.

[105] Wolfersdorf M, Straub R, Barg T, Keller F, Kaschka WP (1999). Depressed inpatients, electrodermal reactivity, and suicide - a study about psychophysiology of suicidal behavior. Arch Suicide Res.

[106] Jandl M, Steyer J, Kaschka WP (2010). Suicide risk markers in major depressive disorder: a study of Electrodermal activity and event-related potentials. J Affect Disord.

[107] Crowell SE, Beauchaine TP, McCauley E, Smith CJ, Stevens AL, Sylvers P (2005). Psychological, autonomic, and serotonergic correlates of parasuicide among adolescent girls. Dev Psychopathol.

[108] Thorell LH, Wolfersdorf M, Straub R, Steyer J, Hodgkinson S, Kaschka WP (2013). Electrodermal hyporeactivity as a trait marker for suicidal propensity in uni- and bipolar depression. J Psychiatr Res.

[109] American Psychiatric Association, APA. Diagnostic and statistical manual of mental disorders, fourth edition: DSM-IV-TR®: American Psychiatric Association; 2000.

[110] American Psychiatric Association, APA (1980). Diagnostic and statistical manual of mental disorders.

[111] ICD-9-CM: International Classification of Diseases, 9th Revision, Clinical Modification. Salt Lake City, Utah: Medicode, 1996. Print.

[112] The ICD-10 Classification of Mental and Behavioural Disorders: Clinical Descriptions and Diagnostic Guidelines. Geneva: World Health Organization, 1992. Print.

[113] Spitzer RL, Endicott J, Robins E (1978). Research diagnostic criteria: rationale and reliability. Arch Gen Psychiatry.

[114] Feighner JP, Robins E, Guze SB, Woodruff RA, Winokur G, Munoz R (1972). Diagnostic criteria for use in psychiatric research. Arch Gen Psychiatry.

